# Effects of a video game intervention on symptoms, training motivation, and visuo-spatial memory in depression

**DOI:** 10.3389/fpsyt.2023.1173652

**Published:** 2023-08-24

**Authors:** Moritz Bergmann, Ines Wollbrandt, Lisa Gittel, Eva Halbe, Sarah Mackert, Alexandra Philipsen, Silke Lux

**Affiliations:** Department of Psychiatry and Psychotherapy, University of Bonn, Bonn, Germany

**Keywords:** cognitive training, video gaming, major depressive disorder, depressive symptoms, training motivation, visuospatial memory

## Abstract

**Background:**

People with Major Depressive Disorder (MDD) often experience reduced affect, mood, and cognitive impairments such as memory problems. Although there are various treatments for MDD, many of them do not address the cognitive deficits associated with the disorder. Playing 3D video games has been found to improve cognitive functioning in healthy people, but it is not clear how they may affect depressed mood and motivation in people with MDD. The aim of this study was to investigate whether a six-week video game intervention leads to improvements in depressed mood, training motivation, and visuo-spatial (working) memory functions in patients with MDD.

**Methods:**

A total of 46 clinically depressed individuals were randomly assigned to one of three groups: an experimental “3D video gaming” group (*n* = 14) which played a video game, an active control group (*n* = 16) which trained with a computer program “CogPack,” and a treatment-as-usual group (*n* = 16) which received a standard clinical treatment including psychotherapy and/or pharmacotherapy. Participants performed a neuropsychological assessment, including self-report questionnaires asking for depressive symptoms, training motivation, and visuo-spatial (working) memory functions before and after the training intervention.

**Results:**

Regarding depressive symptoms, a significant decrease in the proportion of participants who showed clinical levels of depressive symptoms as measured by the Beck Depression Inventory was only found in the 3D video gaming group. Additionally, mean motivational levels of performing the training were significantly higher in the 3D video gaming group when compared with the active control group. Moreover, whereas the 3D Video Gaming group only significantly improved on one visuo-spatial memory test, the active control group improved in all visuo-spatial memory functions. The 3D video gaming group did not perform significantly better than the CogPack group, and the TAU group.

**Conclusion:**

Besides a standalone cognitive training, the current findings suggest that cognitive trainings using a video game have potential to increase subjective well-being, show higher levels of training motivation, and lead to improvements in visuo-spatial (working) memory functions in MDD. However, given the mixed and unblinded nature of this study, the results should be interpreted with caution. Further research with larger samples and follow-up measurements is needed.

## 1. Introduction

The study of the human mind and behavior can be subdivided into two broad categories: the *cognitive* – “how we know the world“- and the *affective* – “how we feel about it.” However, classification into these broad categories is difficult, as both categories have been shown to interact and depend on each other (1). When examining the consequences of pathological changes in these two broad categories, it has been found that cognition can affect emotional well-being and, conversely, that altered emotional well-being can also affect cognition (1). Major depressive disorder (MDD) is one example of a mental health disorder that is commonly associated with affective symptoms such as a low mood, low motivation, and cognitive impairments such as poorer memory functions (2).

Diagnosis and treatment of MDD traditionally focuses on affective symptoms. However, it is now widely acknowledged that MDD is often also accompanied by cognitive dysfunctions (3, 4). Despite decades of research, there are still significant unmet needs and gaps in knowledge that fundamentally impact current therapeutic strategies. In particular, the lack of effective treatments that can rapidly attenuate cognitive and affective symptoms and the lack of more individualized treatment approaches demonstrate very clearly that there has been only little progress in the treatment of cognitive deficits in MDD in recent years. Furthermore, in supposedly remitted patients with MDD, residual cognitive impairments compromise everyday life and interpersonal functioning and increase the risk of a recurrence of depression and comorbidities (5, 6). These persistent patterns of cognitive impairments in remitted depression, notably in the domains of executive functions, attention, and memory suggest that it may serve as a mediator between MDD and poor functional outcomes and explain difficulties many patients face in everyday life (7, 8). The decreased drive and motivation, as typically seen in MDD, further complicates the feasibility of various treatment methods in depressed individuals (9).

With advances in technology leading to more complex, engaging, and immersive video games, recent research has focused on investigating their therapeutic potential (10). Studies with healthy participants have gradually established that playing video games benefits a multitude of cognitive functions, including (visuo-spatial) memory abilities (11–14). However, the existing literature on the effects of video games appears quite heterogeneous. While many publications do not draw on a consistent definition of “video games,” some studies and meta-analyses focus on a narrowly defined set of video games, for example “action video games” (AVGs). AVGs refer to a set of games that are characterized by a fast pace, a high degree of clutter or distraction as well as a high degree of motor load (11). Since AVGs engage multiple cognitive domains at the same time, they have gained increased attention as an intervention for cognitive enhancement. Previous cross-sectional studies suggest that habitual AVG-players demonstrate faster attention allocation, better cognitive control, enhanced mental rotation abilities, and improved spatial skills compared to individuals with little to no video game experience (15–17). Similarly, a large body of longitudinal intervention studies indicates improvement in cognitive performance in naive gamers following an AVG training period in comparison to a control group, thus providing evidence of a causal relationship between AVG play and improvement in cognitive functioning (11, 18–20). Additionally, Clemenson and Stark (20) provided evidence that playing a 3D video game can promote hippocampal plasticity, which consequently led to an enhancement in hippocampus associated cognitive functions, such as visuo-spatial memory (20). They examined naïve gamers and compared the performance of participants who played a complex 3D video game (“Super Mario World”) with the performance of participants who played a simpler 2D video game (“Angry Birds”). Results indicated improved mnemonic discrimination as well as improvements on a virtual water maze task only in the complex 3D video game group. These findings are consistent with the idea that exploration of a visually stimulating virtual environment can act as a form of artificial environmental enrichment.

While the studies mentioned above included samples of healthy adults, recent work has also examined the effects of video game interventions on cognition in participants with MDD. Kühn and colleagues (20) recruited 68 clinically depressed individuals who either played a fast-paced action video game for 6 weeks or were part of a waitlist control group (20). At the end of the intervention, patients in the video game group reported significantly higher subjective cognitive abilities as well as reduced rumination, and they displayed improved cognitive flexibility compared to patients in the waitlist control group. A recent systematic review by Ruiz et al. (21), the authors concluded that video game-based interventions can be effective in ameliorating clinical symptoms of depression (21). However, as the total number of studies testing video game-based therapies on depressed participants was rather small (*n* = 13) and the studies showed a high degree of heterogeneity in terms of applied methods, only precautious conclusions can be drawn.

To the best of our knowledge, so far, no longitudinal study with patients suffering from MDD has implemented a video game intervention as a method to evaluate depressive symptoms, motivational aspects, and cognitive functions in one study design. In addition, no study including depressed participants compared a video game training group with an active control group and a treatment-as-usual group, in which all of whom received parallel regular hospital treatment including psychotherapy and/or pharmacotherapy. Therefore, in the present study, we used the 3D platform game “Super Mario Odyssey on a “Nintendo Switch” console, which requires participants to navigate within 3D environments while relying on processes that depend on the hippocampus and thus potentially influence performance on hippocampally mediated memory tasks, specifically visuo-spatial memory. The present study also includes an active control group that trained with the computerized cognitive training program “CogPack.” Additionally, we included a treatment-as-usual group of participants with MDD, who besides their regular treatment including psychiatric medication and psychotherapy did not receive a specific cognitive training intervention during this study.

Given that participants in the video game and active control group underwent a cognitive intervention, we expect that both groups would benefit from the training procedures and indicate better scores on visuo-spatial memory tests during post-test. Additionally, we predict that the 3D video gaming group will show larger improvements in depressive symptoms and visuo-spatial memory performance when compared with the two comparison groups. We also expect that the 3D video gaming group will show higher motivational training scores when compared to the CogPack group.

## 2. Materials and methods

### 2.1. Participants

The study received ethical approval from the University of Bonn medical faculty ethics committee (protocol number: 326–18) and written informed consent was obtained from all participants. Participants were recruited via the Department of Psychiatry and Psychotherapy at the University Hospital Bonn, which included either inpatients or day-care patients from the clinical wards. To be eligible for the study, participants had to be between 18 and 65 years of age and fulfill DSM-5 criteria (acquired by use of the Mini-DIPS structured clinical interview) for a Major Depressive Disorder (MDD) (22, 23). Individuals were excluded from the study if they had one of the following comorbid disorders of schizophrenia, psychosis, bipolar disorder, or substance abuse disorder, identified using the Mini-DIPS (23). Other exclusion criteria pertained to individuals who had a severe neurological disorder (e.g., history of stroke), or who received electroconvulsive therapy (ECT) at the time of the study. Additionally, participants who indicated a high level of leisure video game usage, or who had prior experience with the video game “Super Mario Odyssey” were excluded from taking part in the study. Participants were fully informed of the procedure and their possibility to withdraw at any time. Participants did not receive any monetary incentive for their participation.

### 2.2. Study design

The study design is a three-arm randomized trial with two measurement timepoints. The three groups compared are: (1) an experimental video game training group that trained using the 3D platform video game Super Mario Odyssey on a Nintendo Switch (3D video gaming group, *n* = 14), (2) an active control group (CogPack, *n* = 16), and (3) a treatment-as-usual group (TAU, *n* = 16). Both treatment groups received three training sessions per week for six weeks, for a total of 18 sessions. The sessions took place individually and lasted for 45 min each. Outcome parameters were evaluated at baseline (T0) and immediately after the 6-week intervention (T1). The primary outcome parameters were changes in the proportion of participants with depressive symptoms, mean training motivation values, and visuo-spatial (working) memory functioning measured using neuropsychological tests.

### 2.3. Procedure

Before the start of the intervention period, all participants were informed about the study and provided written consent. Participants completed self-report questionnaires and a 120-min neuropsychological assessment, including questions about demographics, medical history, video game usage and experience, and cognitive functioning. Participants were then randomly assigned to one of three groups: the experimental group (3D video gaming group), the active control group (CogPack), or the treatment-as-usual (TAU) group. In the 3D video gaming group, participants played the action-adventure game “Super Mario Odyssey” on the Nintendo Switch console while receiving tasks at irregular intervals to actively orient themselves within the in-game environments. These tasks were included to ensure that participants would actively orientate themselves within the in-game environments. In the CogPack group, participants trained with the computerized cognitive training program “CogPack” Version 8.8, which includes exercises for attention, memory, visuo-motor skills, linguistic skills, and mathematical skills. The TAU group received regular treatment in one of our hospital wards, including psychotherapy and/or pharmacotherapy. Six weeks after the pre-assessment, all participants completed a post-test with parallel versions of the pre-assessment questionnaires and neuropsychological tests.

### 2.4. Neuropsychological tests and questionnaires

An overview of all neuropsychological tests and questionnaires used in this study can be found in the Supplementary Table S1. This battery also includes neuropsychological tests covering attention, executive functioning, language production, and visuo-spatial (working) memory functions. This test battery is a standard battery used for all participants in the hospital. However, to answer the research questions of this study only the following tests and questionnaires were evaluated in detail.

#### 2.4.1. Becks depression inventory (BDI-II)

The BDI-II consists of 21 items measuring the severity of depression during the past two weeks.

Each item is scored on a value from 0 to 3 (e.g., 0 = I do not feel sad, 1 = I feel sad, 2 = I am sad all the time and I cannot snap out of it, 3 = I am so sad and unhappy that I cannot stand it); (24). Using total scores, the severity of the depression can be categorized into four levels: minimal (0–13), mild (14–19), moderate (20–28), and severe (29–63).

#### 2.4.2. Training motivation

The training motivation was assessed in all training groups at the end of every training week. Participants were asked to answer the question “How motivated were you to complete and perform the cognitive training?” The question is scored on a value from 0 to 6 (0 = not motivated, 6 = highly motivated). Since participants in the TAU group did not undergo any training intervention, they did not receive the weekly assessments of training motivation.

#### 2.4.3. Wechsler memory scale – block tapping (WMS-block tapping)

The WMS-Block Tapping (25) was used to assess participants’ visuo-spatial short-term working memory. The test consists of nine identical blocks randomly positioned on a flat surface. The experimenter taps a predetermined sequence of blocks while the participant watches. Afterwards, the participant repeats the tapping as presented by the experimenter. In the backwards version, the participant is asked to repeat the tapping order in reverse. The sequence starts out simple and gradually becomes more difficult as the number of blocks tapped increases. Each correctly reproduced sequence is scored with one point and a total of 26 points can be achieved (combined forward and backward versions).

#### 2.4.4. Brief visual memory test – revised (BVMT-R)

The BVMT-R (26) was used to assess the participants’ visuo-spatial learning and memory abilities. During three consecutive ten seconds trials, the participant was asked to remember as many details of six figures arranged in a 2 × 3 matrix as possible. Subsequently, after each trial, the participant was asked to draw the figures from memory in the correct location and as accurately as possible on a sheet of paper. For every drawn figure, the participant could receive a maximum score of two points. One point for the correct location and another one for an accurate sketch, resulting in a total maximum of twelve points per trial and a maximum total recall of 36.

### 2.5. Data analysis

IBM SPSS Statistics for Windows (Version 28 Armonk, NY: IBM Corp) was used to analyze the data. We report significant effects at a threshold of *p* < 0.05. Primary outcome parameters were the proportion of participants who scored a total score above 13 on the Beck Depression Inventory (BDI-II) questionnaire, training motivation scores, and scores on neuropsychological tests measuring visuo-spatial (working) memory functions (BVMT-R, WMS-Block-Tapping). A non-parametric McNemar test was used to evaluate changes in the proportion of patients with a BDI-II score above 13 (indicating mild depressive symptomatology) between pre- and post-measurements in the three treatment groups. For this test participants who scored above 13 on the BDI-II and participants who scored below 13 were binary coded, with “1” representing a BDI-II score above 13 and “0” representing a score below 13. Multiple repeated-measures mixed ANCOVAs that included time (T0 and T1) as a within-subjects factor and group (3D video game, CogPack, or TAU) as a between-subjects factor were conducted to assess the effects of the interventions on neuropsychological tests. In cases where a significant main effect of time was found, further paired t-tests were performed for each treatment group. Although no significant variations in age and gender were observed among the groups studied, these variables were incorporated as covariates in accordance with the findings of Wang et al. (14), who posited that age and gender may exert a moderating influence (14). For the BVMT-R, the total scores of the three trials were calculated. To reach a normal distribution, the scores of the forward and backward versions of the WMS Block-Tapping Test were combined to create a single variable (total sum). For all variables, to correct for multiple comparisons, Bonferroni-Holm corrections were applied. An ANCOVA was used to evaluate differences in mean training motivation scores between the two treatment groups at six-time points (T1-T6), including age and sex as covariates and group membership as a fixed factor. Additionally, effect sizes were estimated using (partial) eta-squared for ANCOVAs and Cohen’s d for paired t-tests, with values of 0.2, 0.5, and 0.8 indicating small, medium, and large effects, respectively. Correlations between pre-and post-tests were corrected for when calculating the effect sizes for paired t-tests (27). Using the software “G-Power” (28), a total sample size of 48 participants was calculated. As similar studies have shown that a medium effect size of 0.30 was sufficient to detect a significant difference between the groups, this was entered into the program in combination with a power-level set to 0.95, and an alpha level of 0.05. To account for potential drop-outs, 54 participants were recruited for this study.

## 3. Results

### 3.1. Participant demographics

**Figure 1:**
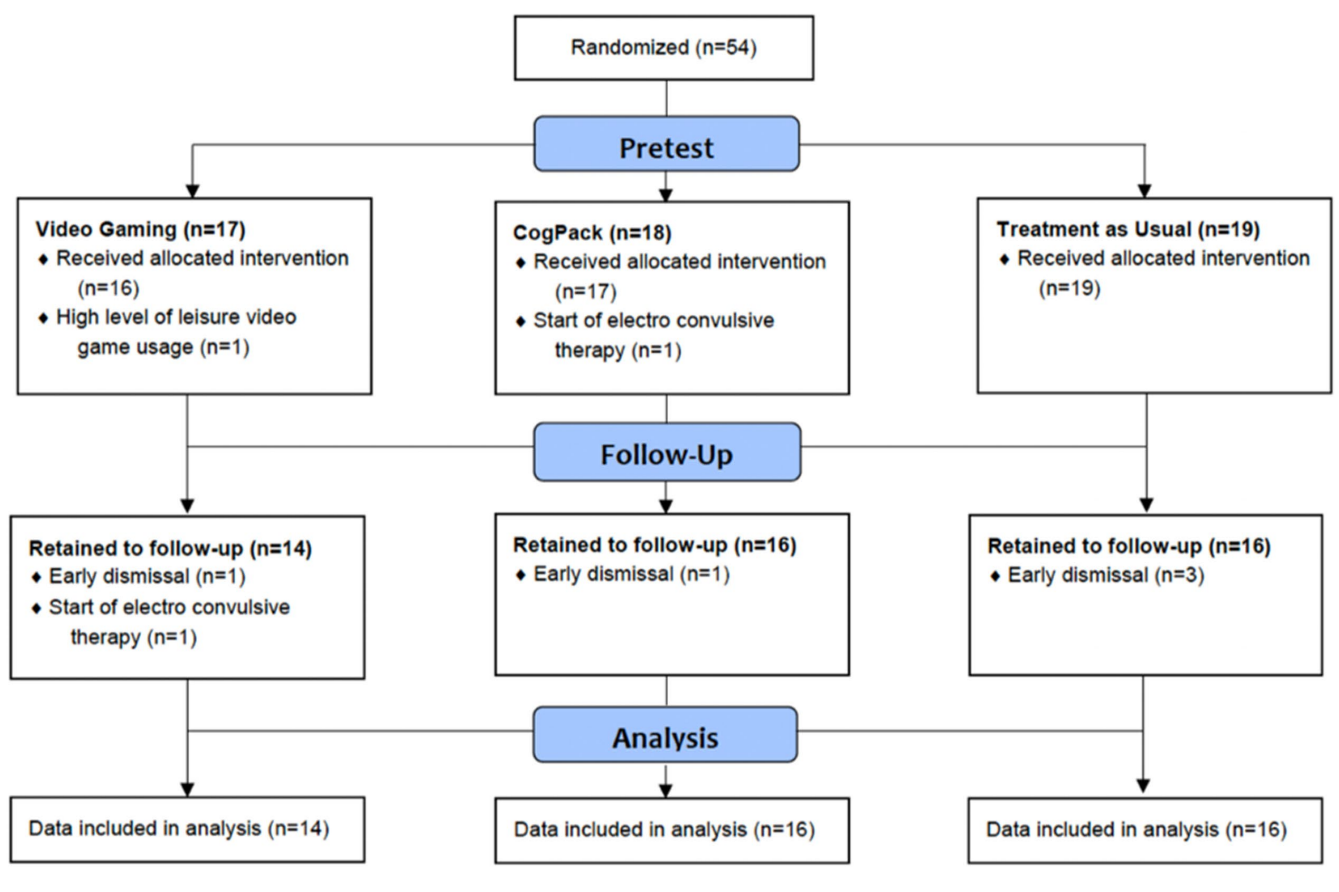
Flowchart indicating participant participation in the randomized controlled trial.

Fifty-four participants that met our exclusion and inclusion criteria took part in the present study. Of these, 46 participants (22 females/23 males; mean age ± SD: 40.7 ± 14.9 years) successfully completed the assessments and interventions. Eight participants were excluded due to an early dismissal (*n* = 5), the start of ECT treatment (*n* = 2), or a high level of leisure video game usage (*n* = 1; see Figure 1). Demographic characteristics of the patients in the three groups at pretest can be found in Table 1. There were no significant differences in age, gender, educational qualifications, BDI-II scores, number of training sessions, number of depressive episodes, video game experience, medication-intake, number of comorbidities, or usage of electronic devices during pretest.

**Table 1:** Pretest characteristics of participants.

Characteristics	3D Video Gaming (*n* = 14) Mean ± SD	CogPack (*n* = 16) Mean ± SD	TAU (*n* = 16) Mean ± SD	Statistic^a^	*p*-value^b^
Age (years)	36.29 ± 11.74 (21–57 years)	44.06 ± 17.06 (18–63 years)	41.13 ± 14.93 (18–65 years)	*F*_(2,43)_ = 1.03	0.36
Sex (f/m)	4/10	11/5	7/9	χ^2^ = 4.99	0.08
Qualification (CS/SE/A)^c^	2/1/11	0/3/13	0/2/14	χ^2^ = 5.43	0.25
BDI-II	28.29 ± 6.49	22.62 ± 5.22	19.31 ± 4.64	χ^2^ = 1.55	0.69
Number of training sessions	16.79 ± 1.89	15.19 ± 2.66	-	*F*_(2,43)_ = 1.87	0.07
Video gaming experience	9.79 ± 5.16	8.06 ± 4.67	10.50 ± 4.40	*F*_(2,43)_ = 1.11	0.34
Antidepressant medication (no/yes)	1/13	2/14	1/15	*F*_(2,43)_ = 0.46	0.80
Sedative medication (no/yes)	10/4	13/3	14/2	*F*_(2,43)_ = 1.23	0.54
Number of psychiatric comorbidities	1.28 ± 0.47	1.25 ± 0.77	1.13 ± 0.34	*F*_(2,43)_ = 0.35	0.71
Usage of electronic devices	10.00 ± 5.60	6.13 ± 5.07	7.44 ± 6.85	*F*_(2,43)_ = 1.65	0.21

*n*, number of participants; SD, Standard Deviation; TAU, treatment as usual. ^a^One-way ANOVA or chi-square tests were used. ^b^Sign. alpha level at *p* < 0.05. ^c^CS, comprehensive school; SE, secondary education; A, A-level.

### 3.2. Primary outcomes

#### 3.2.1. Rates of depression

**Figure 2:**
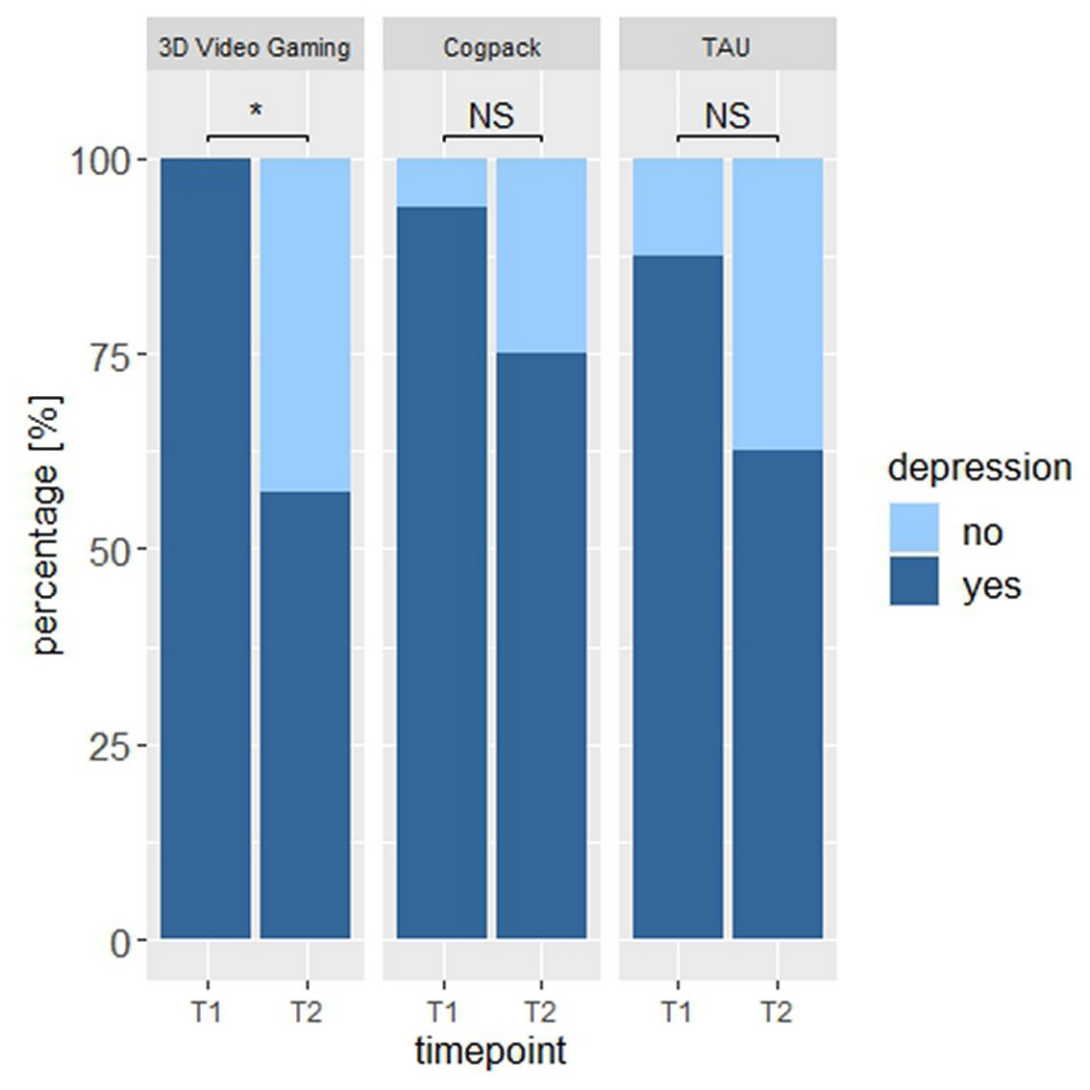
The proportion of participants that showed depressive symptoms (BDI-II ≥ 13) at pre- and post-assessment. * an Asterix indicates a sign. Value of *p* at alpha level 0.05. NS, non-significant.

An exact McNemar test found a statistically significant difference in the proportion of participants with depressed symptoms (BDI-II ≥ 13) between pre-and post-intervention only in the 3D video gaming group (percentage of depressed participants at pre-measurement = 100, and at post-measurement = 57; *p* = 0.03; post-intervention raw-score mean = 16.14), but not in the CogPack group (percentage of depressed participants at pre-measurement = 94, and at post-measurement = 75; *p* = 0.25; post-intervention raw-score mean = 22.5) or TAU group (percentage of depressed participants at pre-measurement = 75, and at post-measurement = 63; *p* = 0.13; post-intervention raw-score mean = 19.44; see Figure 2). The scores would not survive when correcting for multiple comparisons using the Bonferroni-Holm method. Still, we found evidence against the null hypothesis that there was no effect of cognitive training on depressive symptoms in the 3D video gaming group and conclude that the proportion of patients with depressive symptoms significantly decreased over time only in the 3D video gaming group.

#### 3.2.2. Levels of training motivation

**Figure 3:**
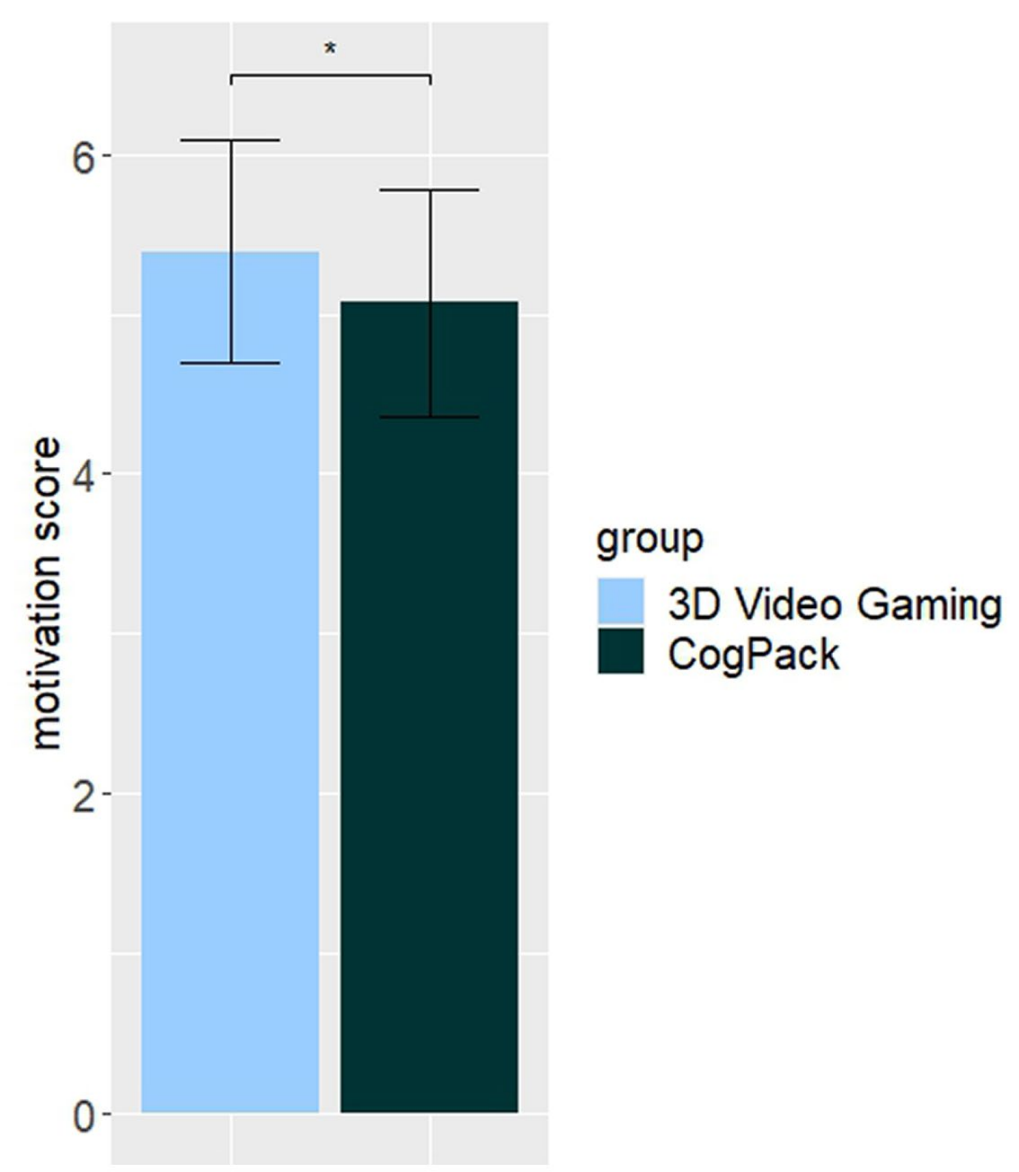
Subjective assessment of mean motivation to perform the cognitive training. Note. Error bars indicate the 95% confidence intervals. * an Asterix indicates a sign. Value of *p* at alpha level 0.05.

Using an ANCOVA we compared mean motivational levels across the two intervention groups over the training period of six weeks. When controlling for effects of age (*p* = 0.03) and sex (*p* = 0.049), we found a significant effect of group [*F*(3,25) = 6.49, *p* = 0.02, η_p_^2^ = 0.20], indicating a higher motivation to perform the training in the 3D video gaming group (*M* = 5.39, SD = 0.70) compared to the CogPack group (*M* = 5.07, SD = 0.71; see Figure 3; for the individual timepoints see Supplementary Figure S1). We therefore reject the null hypothesis that there is no difference in motivational scores across the two intervention groups and conclude that the overall motivation to perform the cognitive training was higher in the 3D video gaming group compared to the CogPack group.

#### 3.2.3. Visuo-spatial (working)-memory

**Figure 4:**
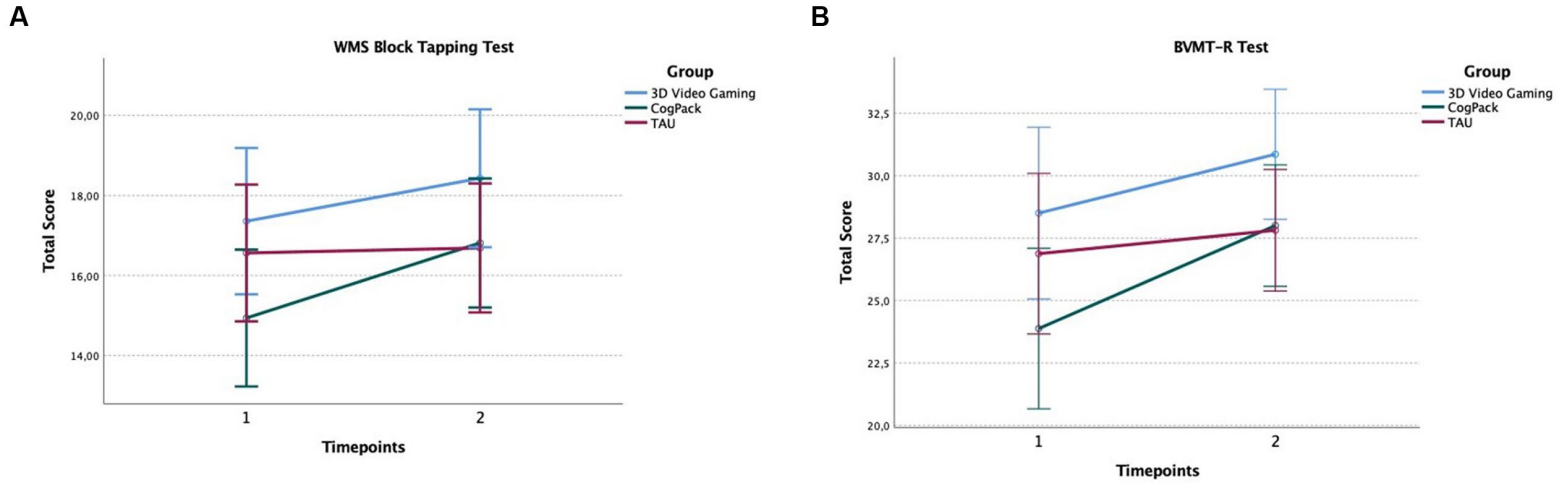
Performances in the WMS Block Tapping Test **(A)** and BVMT-R Test **(B)** at pre- and post-assessment. Error bars indicate the 95% confidence intervals.

When adjusting for the effects of age and gender in the BVMT-R and WMS Block Tapping test, the group x time interaction did not reach the statistical level of significance (BVMT-R: *F*(2,41) = 2.87, *p* = 0.07, η_p_^2^ = 0.12; WMS Block Tapping: *F*(2,41) = 1.89, *p* = 0.16, η_p_^2^ = 0.08). However, we found significant main effects of time for the variables BVMT-R [*F*(2,41) = 16.88, *p* = 0.00] and the WMS Block Tapping Test [*F*(2,41) = 8.88, *p* = 0.005; see Figures 3, 4]. Further paired t-tests comparing pre- and post-intervention performances indicated significant improvements over time for the BVMTR variable in the 3D video gaming group [*t*(13) = −2.18, *p* = 0.048, *d* = 0.58] and CogPack group [*t*(15) = −3.72, *p* = 0.002, *d* = 0.93], but not for the TAU group [*t*(15) = −1.01, *p* = 0.33, *d* = 0.25]. For the WMS Block Tapping Test, we found significant improvement over time only in the CogPack group [*t*(15) = 3.64, *p* = 0.002, *d* = 0.90], but not in the 3D video gaming group [*t*(13) = 1,43, *p* = 0.18, *d* = 0.38] or the TAU group [*t*(15) = 0.23, *p* = 0.81, *d* = 0.06]. After applying a Bonferroni-Holm correction for multiple testing, only the *p*-values of the CogPack group would survive.

## 4. Discussion

Recently, cognitive trainings in the form of video games have gained increasing interest as an additional treatment option in psychiatric disorders. However, due to the rather heterogeneous methodologies of past studies, effects on depressed patients remain largely unclear. To our knowledge, this is the first randomized controlled study that included a treatment-as-ususal group of inward patients receiving medical treatment and psychotherapy as well as an active control group that received cognitive training with a standard computerized cognitive training program “CogPack.” Therefore, the aim of the present study was to examine the effects of a six-week 3D video game intervention on symptoms of depression, training motivation, and visuo-spatial (working) memory performance in patients with MDD.

### 4.1. Effects on rates of depression

The results showed that the proportion of participants with BDI-II scores above the threshold was only significantly lower at post-test in the group that received video game training, but not in both control groups. These findings suggest that the addition of video game training to regular treatment including psychotherapy and/or pharmacological treatment can lead to reductions in depressive symptoms. This is consistent with previous research indicating that videogame play can reduce depression within four weeks when compared to a control group (29). A core symptom that is common in depression is an increased level of rumination, a type of repetitive negative thinking pattern that has been linked to prolonged and exacerbated emotional states and impaired cognitive function (29–32). The mechanism behind the reduced depressive symptoms seen in the 3D video gaming group may be related to a decrease in rumination. A recent study by Kühn et al. (20) found that depressed patients who participated in a six-week video game intervention had significantly lower levels of rumination compared to a waitlist control group (20). Although this current study did not specifically assess rumination, the improvement in depressive symptoms observed in the 3D video gaming group suggests that reductions in rumination may have contributed to the observed effect. However, it should be noted that the 3D video gaming group had a higher proportion of participants with BDI-II scores above the threshold at pretest, which may have reduced the statistical power of the study. Despite this limitation, the results suggest that video game training may be a useful adjunctive treatment option for reducing depression in patients with MDD.

### 4.2. Effects on training motivation

It was hypothesized that participants in the 3D video gaming group would report greater motivation to complete and perform the intervention. The results showed that the mean motivational values were higher in the video game training group, which is consistent with previous research indicating that video games may be more motivating than other types of cognitive interventions (33). Video games have one clear advantage over classical cognitive training software: they are created to be fun and entertaining and have been found to evoke positive emotions, thus increasing the player’s emotional investment (34). The exploration of characters, actions, and environments within video games is inherently interesting and creates a suspense which might increase the likelihood of gameplay and therefore compliance with treatment (35). In addition, video games often offer rewards for good performance in terms of positive feedback or incentives that encourage players to continue playing. Additionally, video games may provide a sense of control over one’s environment and outcome, which can be particularly beneficial for depressed individuals who often feel a lack of control over their lives. The ability to make choices and experience the consequences of those choices in a safe and controllable environment of a video game could be empowering and motivating. Based on the self-determination theory, Przybylski et al. (36) have proposed their motivational model of video game engagement, stating that video games can provide players with experiences that satisfy universal needs of competence, autonomy, and relatedness, thus offering the potential to enhance intrinsic motivation (36). For instance, modern video games support *autonomy* by providing players with a multitude of in-game choices including what missions they choose or how their characters appear. Additionally, skill-graded challenges and positive feedback promote experiences of *competence* while the need for *relatedness* is satisfied by technologies such as voiceover internet protocol communication that allows players to cooperate with geographically remote peers. Since MDD patients often experience low mood in combination with a loss of interest, the highly intrinsically motivating characteristics of video gaming might offer new treatment possibilities that are especially suited for this patient group by increasing motivation and rates of treatment adherence. However, it is worth noting that both the 3D video gaming and CogPack groups had very high mean motivational scores (*M* = 5.39 and *M* = 5.07, respectively), which may indicate ceiling effects. It is possible that the expectations of cognitive enhancement associated with both types of training may have influenced participants’ motivation, regardless of the specific nature of the intervention. In future studies, it may be useful to adapt the motivation measurement to specifically address the motivating factors of the intervention method in order to more accurately assess the effect of the video game or training program on motivation. This could be done according to the self-determination theory, in which various questions that ask for autonomy, competence, and relatedness could be included (36). In addition, examining in-game performance and its relationship to motivation could provide further insight into the effects of video game training on motivation. For example, Rieger et al. (37) reported that in-game success was predictive of post-game mood adjustment, and frustration during gameplay may decrease motivation and potentially undermine the effects of the intervention on cognition (37). Hence, future work may seek to log performance data to evaluate potential associations between performance in the video game to motivational aspects.

### 4.3. Effects on visuo-spatial (working) memory functions

Results of this study indicate significant improvements over time in visuo-spatial (working) memory for both training groups after the intervention. However, there was no significant interaction effect for any of the dependent variables, indicating that the 3D video gaming group did not perform significantly better at post-assessment than the control groups. The CogPack training group showed significant improvements on the Brief Visuo-spatial Memory Test-Revised (BVMT-R) and the Wechsler Memory Scale Block Tapping Test (WMS Block Tapping Test), while the 3D video gaming group only showed a significant effect on the BVMT-R. The small sample size (*n* = 14) in the 3D video gaming group, which already displayed relatively high baseline scores on the WMS Block Tapping Test at pretest, may have reduced the likelihood of finding significant improvements on this test.

Our results point in the direction of findings from previous studies demonstrating that the exploration of a vast three-dimensional game environment can lead to hippocampus-associated improvements in cognition, namely visuo-spatial memory. Specifically, Kühn et al. (38) were able to demonstrate that playing the 3D platform game “Super Mario 64” for 30 min per day for two months can lead to significant gray matter increases in the right hippocampal formation, right dorsolateral prefrontal cortex, and bilateral cerebellum when compared to a passive control group (38). Additionally, Clemenson and Stark (39) provided evidence that playing a complex 3D video game can stimulate the hippocampus, leading to an enhancement in hippocampus-associated cognition (39). One possible explanation for the improvements over time in both training groups might pertain to the fact that the computerized cognitive training program CogPack that was used by participants in the active control group does also involve exercises for visuo-spatial memory skills, potentially undermining any effect of the video game training. Indeed, it has been established that studies with active control groups tend to obtain less favorable results compared to studies with only passive controls, possibly because participants in the experimental group as well as the active control group would have expectations regarding the training outcome (40). Therefore, since the two training interventions displayed overlap regarding the addressed cognitive domains, the training effect of the video game might have been underestimated in the present study. Yet, we propose that future studies implement the use of control group(s) where participants are active and challenged, as it leads to a more useful assessment of the efficacy of an intervention and offers the possibility to rule out potential confounds.

Videogaming as well as computerized cognitive training appears beneficial for a multitude of cognitive functions, as has been established by previous research in healthy populations (11, 41) and individuals with major depressive disorder (22, 42, 43). However, only a few studies have directly compared video games and cognitive training programs regarding their impact on cognition. In a study by Perrot et al. (44), 36 participants either trained with the cognitive training game *Kawashima Brain Training* or the video game *Super Mario Bros* for a total training time of 23 h for two months (44). Their results indicate that both training groups demonstrated significant improvements in some aspects of cognition but that the benefits after training with the video game appear broader in nature compared to the benefits resulting from the cognitive training program. Notably, in the present study both groups showed significant cognitive improvements in certain aspects. However, it is important to note that the active control group showed more widespread cognitive benefits. In contrast, the 3D video gaming group only displayed improvements in the BVMT-R and not in the WMS Block Tapping Test, indicating a more selective improvement in visuo-spatial memory functions. As noted earlier, it would have been interesting to measure the expectations regarding the training intervention and its outcome for each group to examine how these expectations might have influenced the training effects. Moreover, in light of non-normal distributions observed in the data of the WMS Block Tapping Test, combined scores of the forward and backward versions were used to enable the use of parametric statistical tests. It should be noted that while the WMS backward subtest assesses both working memory and executive functions, it also requires participants to mentally manipulate the stored information. Conversely, the WMS forward subtest specifically measures visuo-spatial working memory by assessing temporary storage and manipulation of information. Although combining these scores enabled the utilization of a parametric statistical test, there is a possibility that this approach could have resulted in a loss of information. Another direction for future research is to investigate the transfer of training for everyday competencies since video games appear to provide a highly variable environment that involves alternation among a wide range of tasks which could benefit cognitive transfer (44). Additionally, with the use of neuroscientific imaging techniques one could add to the line of research by Kühn et al. (38) and Clemenson and Stark (39) to investigate the effects of video game training and computerized cognitive training on brain structure, specifically hippocampal grey matter and functional neuronal activity patterns in participants with MDD to further examine their potentiality as a therapeutic intervention method (38, 39).

### 4.4. Limitations and future research

A limitation of this study is the small sample size which may have reduced the power of the present design to detect effects. Recruiting this patient population in a clinical sample, however, presents a non-trivial challenge, especially given the intensity and duration of the intervention. Therefore, future work should aim at increasing the sample size in order to achieve more statistical power. Another critical point is the non-blind nature of the intervention, regarding participants and the experimenter likewise. This might have caused expectation and experimenter biases respectively, thus negatively impacting the statistical results. Future studies should address this issue. Furthermore, because all participants received regular clinical treatment in one of our clinical wards, including psychiatric medication and psychotherapy, in parallel with their cognitive training, a change in psychiatric medication or other unforeseen clinical circumstances could also have affected participants’ test results in this study. Thereby, the date of hospitalization also represents a factor, which may have influenced the test results. We tried to keep these factors and hospitalization time-points in our samples as homogeneous as possible. Nevertheless, future studies should try to control for this. Additionally, the duration of the training periods may not have been long enough to conclude whether video games are an effective therapy for the treatment of depression in the long term. Previous intervention studies have typically employed training periods lasting from approximately 4 to 12 weeks, as noted in a recent review (21). Given the heterogeneity of methodologies in previous studies, it is important for future research to also examine the effects of longer interventions (e.g., more than 6 weeks) using video gaming therapy for depressed patients. Moreover, although the observed difference did not reach statistical significance, it is important to note that the 3D video gaming group participated in on average a slightly greater number of training sessions compared to the active control group. This disparity may be attributable to heightened motivational factors associated with the video game training and may have had an impact on the study’s outcomes. Aside from that, regarding visuo-spatial (working) memory functions only significant main effects for time per group were found and these were calculated even in absence of significant group x time interactions. Therefore, one has to be cautious when generalizing the results. As a last point, given that all participants were either inpatients or daycare patients during the course of this study, future work might strive to add a follow-up measurement a few weeks after patients were discharged from hospital to examine whether possible effects persist over time and within participants´ familiar surroundings. This would also allow us to better examine possible transfer effects that the training might bring about.

## 5. Conclusion

In summary, this study is the first randomized controlled study to evaluate the possible effects of a six-week video game intervention on symptoms of depression, training motivation, and visuo-spatial cognition in participants with major depressive disorder (MDD). To validate the test results, this study included two inward active control groups: one that received a standardized cognitive training using the computer software “CogPack” and another that received regular clinical treatment (TAU). Results indicate that after six weeks of training the 3D video gaming group showed a significant decrease in the proportion of participants with clinically significant levels of depressive symptoms by self report and a higher mean training motivation when compared with the active control group. Furthermore, results suggest significant improvements in tasks of visuo-spatial (working) memory performance during post-testing in both training groups, however, the 3D video gaming group demonstrates more selective improvements and does not perform significantly better than the other two groups. Still, these mixed findings suggest that video game training may be a cost-effective and feasible intervention for patients with MDD that can be used in conjunction with regular treatment and therapy. To date, only a few studies compared the effectiveness of video game training with cognitive training programs. As a result, more research is needed, including follow-up measurements and assessments using fMRI, before we fully understand the underlying mechanisms.

## Data availability statement

The raw data supporting the conclusions of this article will be made available by the authors, without undue reservation.

## Ethics statement

The studies involving human participants were reviewed and approved by the local ethics committee of the Medical Faculty of the University of Bonn, Venusberg-Campus 1, E-mail: ethik@ukbonn.de. The patients/participants provided their written informed consent to participate in this study.

## Author contributions

MB mainly conceptualized the study. SL contributed to the conception and design of the study. MB, IW, SM, and LG acquired the data and performed data analyses. EH supported the design of graphs and figures and helped with statistical questions. MB, EH, SL, and AP contributed to data interpretation and discussion of results. MB wrote the first draft of the manuscript. All authors have read and approved the submitted version.

## Funding

This work was supported by the Open Access Publication Fund of the University of Bonn.

## Conflict of interest

AP declares that she served on advisory boards, gave lectures, performed phase-3 studies, and received travel grants within the last five years from MEDICE Arzneimittel, Pütter GmbH and Co KG, Takeda, Boehringer, Janssen-Cilag, and receives royalties from books published by Elsevier, Hogrefe, Kohlhammer, Karger, Oxford Press, Thieme, Springer, and Schattauer.

The remaining authors declare that the research was conducted in the absence of any commercial or financial relationships that could be construed as a potential conflict of interest.

## Publisher’s note

All claims expressed in this article are solely those of the authors and do not necessarily represent those of their affiliated organizations, or those of the publisher, the editors and the reviewers. Any product that may be evaluated in this article, or claim that may be made by its manufacturer, is not guaranteed or endorsed by the publisher.
