# Supplementary material for: Effects of a video game intervention on symptoms, training motivation, and visuo-spatial memory in depression
Source: Front Psychiatry. 2023 Aug 24;14:1173652. doi: 10.3389/fpsyt.2023.1173652 (PMC10484510; doi:10.3389/fpsyt.2023.1173652)
Supplement: Supplementary file 1 [file Table_1.docx]

Supplementary Material

**Effects of a Personalized Video Game Intervention on Symptoms, Training Motivation, and Visuo-Spatial Memory in Depression**

Moritz Bergmann^1*^, Ines Wollbrandt^1^, Lisa Gittel^1^, Eva Halbe^1^, Alexandra Philipsen^1^, Silke Lux^1^

^1^Department of Psychiatry and Psychotherapy, University of Bonn, Bonn, Germany

***Correspondence**

Moritz Bergmann

[Moritz.bergmann@ukbonn.de](mailto:Moritz.bergmann@ukbonn.de)

**Supplementary Table S1**

*Test battery used at pre- and post-assessment*


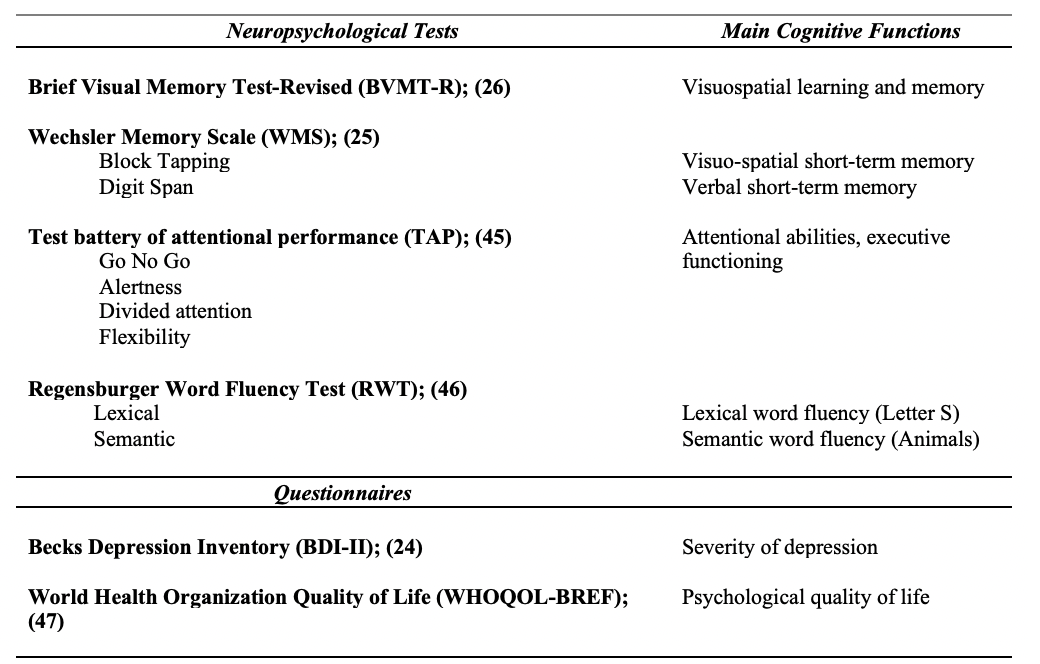


**References**

45. Zimmermann, P., & Fimm, B. (2009). Testbatterie zur Aufmerksamkeitsprüfung (TAP) (2.2). Psytest.

46. Harth, Sandy & Müller, Sandra & Aschenbrenner, S. & Tucha, Oliver & Lange, Klaus. (2004). Regensburger Wortflüssigkeits-Test (RWT). Zeitschrift Fur Neuropsychologie - Z NEUROPSYCHOL. 15. 315-321. 10.1024/1016-264X.15.4.315.

47. The WHQOL Group (1998). Development of the World Health Organization WHOQOL-BREF quality of life assessment. Psychol Med; 28:551-8
