# Supplementary figures and images for: Effects of a video game intervention on symptoms, training motivation, and visuo-spatial memory in depression
Source: Front Psychiatry. 2023 Aug 24;14:1173652. doi: 10.3389/fpsyt.2023.1173652 (PMC10484510; doi:10.3389/fpsyt.2023.1173652)

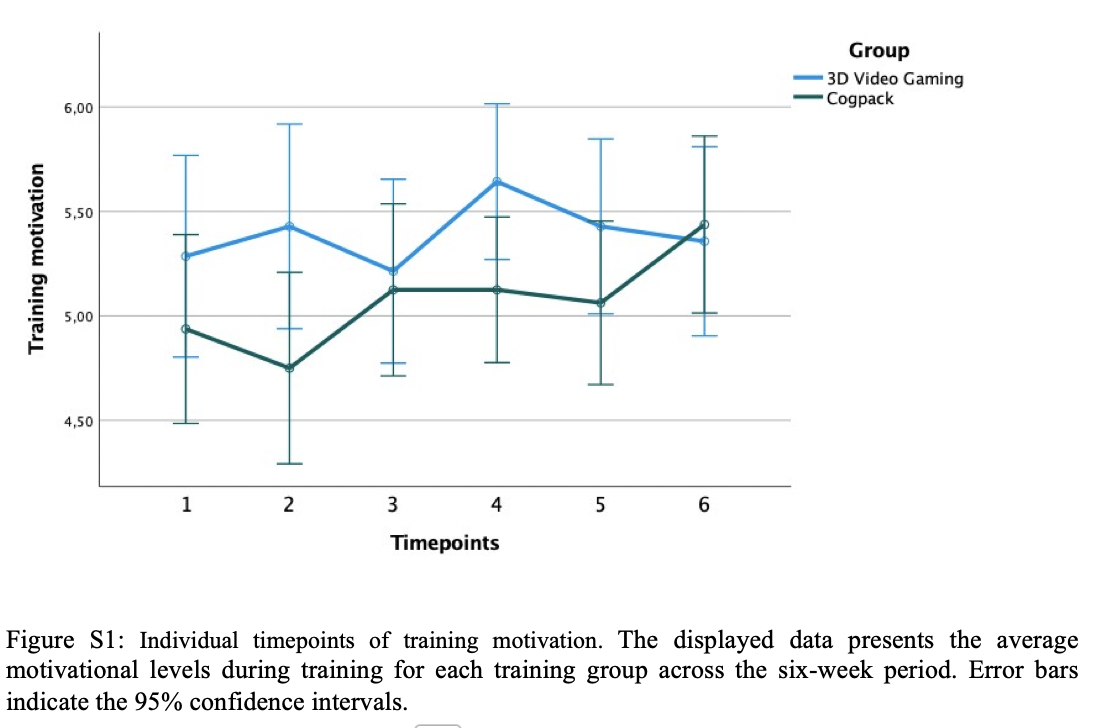

Supplement: Supplementary file 2 [file Image_1.png]
